# Editorial: Distributed networks: new outlooks on cerebellar function, volume II

**DOI:** 10.3389/fnsys.2024.1362963

**Published:** 2024-02-01

**Authors:** Richard Apps, Jimena Frontera, Lisa Mapelli, Thomas Watson

**Affiliations:** ^1^School of Physiology, Pharmacology and Neuroscience, Faculty of Life Sciences, University of Bristol, Bristol, United Kingdom; ^2^Neurobiologie Intégrative des Systèmes Cholinergiques, Département de Neuroscience, Institut Pasteur, CNRS UMR 3571, Paris, France; ^3^Department of Brain and Behavioral Sciences, University of Pavia, Pavia, Italy; ^4^Simons Initiative for the Developing Brain, Centre for Discovery Brain Sciences, University of Edinburgh, Edinburgh, United Kingdom

**Keywords:** cerebellum, motor, cognition, emotion, prediction error, brain disorders, fear

Since the original launch of this Research Topic in 2013, much progress has been made in our understanding of cerebellar contributions beyond the sensorimotor realm. Overwhelming evidence now indicates that the cerebellum is involved in a diverse range of behavioral processes such as decision making, spatial navigation and emotional control. Indeed, it can be argued that the cerebellum is likely involved in all types of behavior, ranging from the control of involuntary reflexes to higher order problem solving.

This second volume brings together findings from the animal, theoretical and clinical fields and includes 18 articles involving 74 authors that explore the latest evidence of cerebellar contributions to motor and non-motor functions (Figure 1). Cerebellar contributions are considered in terms of brain wide networks, information processing, and the clinical consequences that arise when these circuits are disrupted.

**Figure 1:**
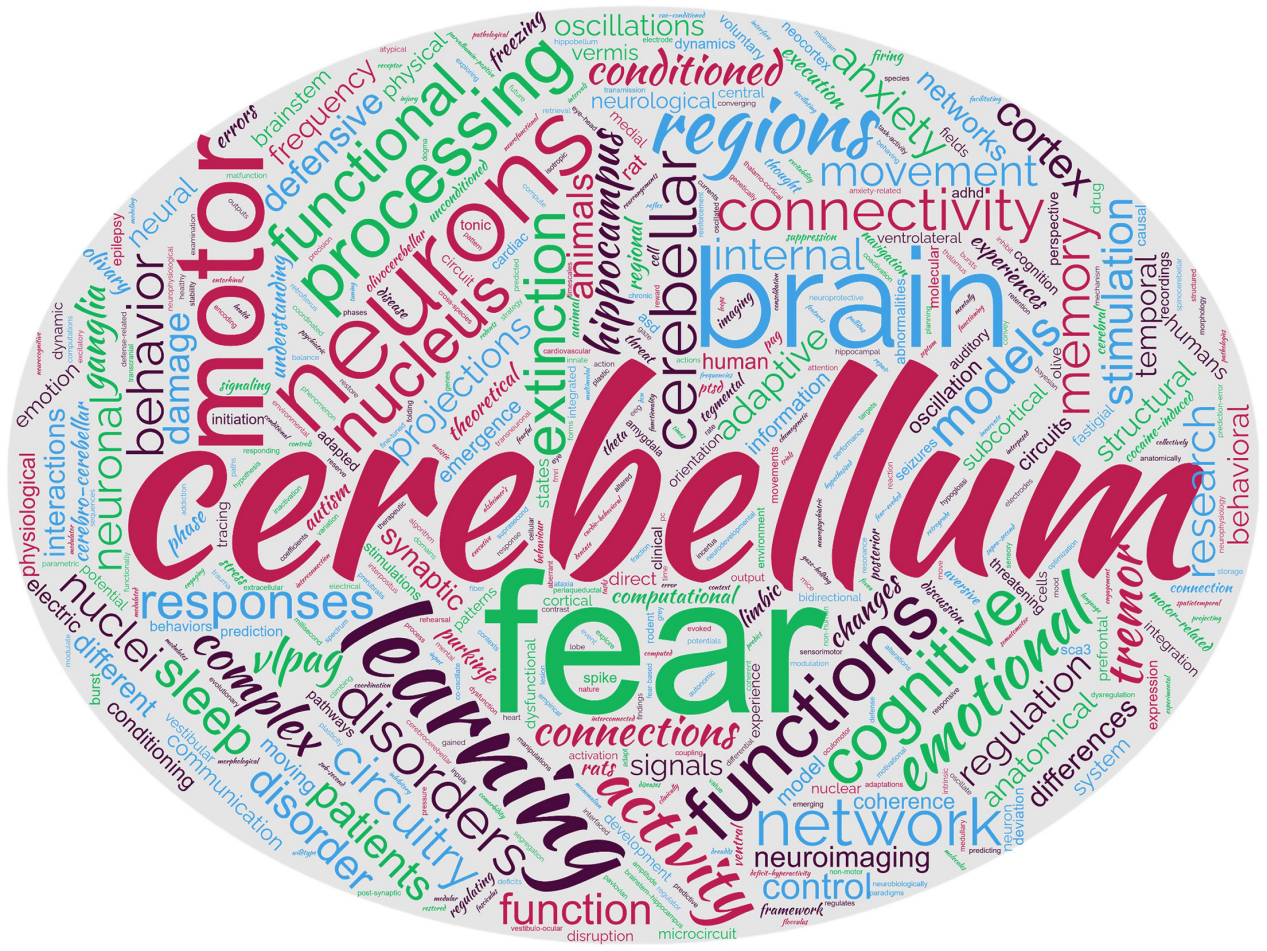
A word cloud based on the abstracts of all 18 articles in this Research Topic. The larger the size of a word the greater the frequency of use. Generated using WordClouds.com.

## Cerebellar contributions to brain wide networks

A cerebellar role in brain wide networks involved in movement control, navigation, cognition and emotional behavior are considered in the present Research Topic. This includes growing evidence that supports bidirectional functional connectivity between the cerebellum and the hippocampus, a brain structure well-known for its role in learning, memory, and spatial processing. A detailed comparison of common network elements between the cerebellum and hippocampus and specialized subnetworks in diverse motor-related processing is reviewed by Henschke and Pakan. This includes an overview of human neuroimaging data, complemented by evidence from animal models that advance our understanding of cerebro-cerebellar interactions during movement execution and motor imagery. Similarly, Froula et al. review recent literature regarding cerebellar-hippocampal interactions (the “hippobellum”) and discuss potential insights and clinical avenues in both healthy brains in terms of spatial navigation and in pathological conditions, such as epilepsy. In the perspective by Cheron et al., cerebellar interactions with the hippocampal network are extended to consider projections to the cerebellum from the brainstem nucleus incertus (NIC). The NIC is related to the vestibulo-ocular reflex and gaze holding and therefore could participate in the hippobellum network by providing eye-head and body movement signals crucial for navigation.

Another important network considered in the current Research Topic is the interconnectivity between the cerebellum and the basal ganglia. The article by Ruigrok et al. presents anatomical results from transneuronal tracing experiments in rats, together with data in mice from the Allen Brain Institute, to provide evidence for basal ganglia interactions with the cerebellum via the inferior olive climbing fiber system. The authors propose that this connection may influence olivary involvement in cerebellar learning, including reward-related behavior.

An increasing body of evidence also indicates that the cerebellum is part of a distributed brain network involved in defensive behaviors elicited by aversive or fearful stimuli. In the review by Neubert da Silva et al. the authors argue that the cerebellum is well placed to integrate the multimodal and highly dynamic processes associated with defensive behavior, including orchestrating motor and autonomic responses. This is linked to a common theme that runs throughout many of the articles contributing to this topic, namely that the cerebellum is involved in prediction error required for the updating of internal models—in the case of defensive responses the cerebellum is thought to predict threat occurrence.

In terms of predictive processes that underlie fear learning and extinction, Desmaison et al. review recent findings that support a role for the cerebellum as a modulator of fear and extinction learning, using prediction error signaling and regulation of fear related thalamo-cortical oscillations. Internal models, prediction error and associated learning and memory processes are explored further in the review article by Doubliez et al. who focus on evidence that the cerebellum in both rodents and humans plays an important role in the extinction of learned defensive responses. They present the anatomical, physiological and neuroimaging evidence that the cerebellum, through its connections with the limbic system, supports the extinction of associatively conditioned fear memory. Omission of the unconditioned stimulus during fear extinction can be considered a sensory prediction error, leading to revision of an internal model that, in turn, leads to a modification in learnt behavior.

Consistent with this proposition, Paci et al. use dynamic causal modeling (DCM) applied to auditory event related potentials recorded in rats during fear conditioning to study effective connectivity between the cerebellar medial nucleus (MCN) and the ventrolateral periaqueductal gray (vlPAG)—the latter is a key node in limbic networks that underpin defensive behaviors such as freezing. Their results provide evidence that communication between MCN and vlPAG increases during extinction, consistent with the cerebellum playing a role in prediction error processes linked to extinction of fear conditioned responses.

A broader role of the cerebellum in learning and memory processes is considered in the review by Gelfo et al.. Namely, that experience-dependent learning leads to neuroprotective effects within the central nervous system (neural reserve) and the evidence that “cerebellar reserve” is important in mitigating the impact of neural damage through accident or disease. The theme of learning and memory is also explored in the review by Jackson and Xu who consider the evidence that the cerebellum participates in off-line memory consolidation during sleep. They point to evidence that suggests that the cerebellum has an important role in shaping the sleep-wake cycle, thereby contributing to memory consolidation processes. In relation to internal models, Jackson and Xu propose that during sleep the cerebellum performs similar computations as in the awake state, updating internal models based on prediction error. However, during sleep this is based on a comparison between simulated motor commands and their virtual sensory consequences.

## Information processing

Cerebellar contributions to information processing have been widely documented in the sub-second domain, where the cerebellar network can provide timing with millisecond precision to coordinate movements. Boven and Cerminara review the evidence that this temporal information processing can be extended to supra second timescales, and thus the control of long-term motor sequences and their planning. They argue that such a role might rely on interactions with the cerebral cortex (such as the prefrontal cortex), and in relation to prediction error, that the cerebellum predicts the desired task outcome and provides feedback to cerebral circuits to coordinate behavior over multiple timescales.

More generally, Ciapponi et al. consider the computational algorithm that underpins cerebellar contributions to behavior. The authors identify some general properties of cerebellar organization, and in line with the review by Neubert da Silva et al., consider how the interplay between segregation and distribution of function in different cerebellar regions might encode the motor, cognitive and autonomic components of a given behavioral response, exemplified by the defensive reaction to a fearful event. From a computational perspective, a cerebellar algorithm working as a forward and inverse controller, provides predictions based on current state and previous memory. However, regional cerebellar variations might adapt the general processing algorithm to specific functions. Including for example, those highlighted by Boven and Cerminara at the supra-second timescale, necessary for motor planning.

## Clinical implications

With regard to the clinical consequences that arise when cerebellar-related brain networks are disrupted, Cundari et al. review cerebellar involvement in three brain disorders: autism, attention deficit hyperactive disorder (ADHD) and spinocerebellar ataxia type 3 (SCA3). They caution that the causal nature of cerebellar contributions to these and other brain disorders remains in its infancy. Nonetheless, regarding autism, Couto-Ovejero et al. bring together anatomical and physiological evidence that links autism to cerebellar contributions to emotional behavior. They argue that the emotional dysregulation observed in autism may be linked to cerebellar deficits, resulting in aberrant encoding of prediction error, and that deficits in internal model calibration could represent a common thread through which we can begin to understand cerebellar contributions to a variety of brain disorders.

Gil-Paterna and Furmark review the evidence that dysfunction in cerebellar contributions to emotional behavior plays a role in post-traumatic stress disorder and related anxiety disorders. Consistent with the animal literature on fear conditioning and extinction, the vermis appears to be the cerebellar region most likely to be involved, though it is clear that further work is required to fully understand the nature of this contribution. On a related theme, Melchor-Eixea et al. explore cerebellar involvement in substance abuse disorder (SUD), which includes emotional, cognitive and motivational components. Their hypothesis and theory article includes preliminary data obtained in rats that chemogenetic modulation of specific regions of the posterior cerebellar vermis can interfere with cocaine-induced place preference without effecting motor performance. This raises the interesting possibility that cerebellar circuits modulate brain networks involved in addiction. If supported by further studies this would open a new avenue for potential treatment of SUD.

Whilst the majority of clinically-oriented studies in this Research Topic are focused on new vistas of cerebellar involvement in non-motor functions, Baumel et al. present results related to essential tremor—a motor disorder linked to cerebellar dysfunction. Single-unit recording from the cerebellar nuclei in freely moving rats in which harmaline-induced tremor was chemically suppressed, show that the suppression of body tremor restores pairwise coherence in neuronal oscillatory activity, suggesting that such activity plays a role in normal motor function.

Finally, recent evidence suggests that cerebellar transcranial alternating current stimulation (ctACS) has promise as a potential treatment for a variety of brain disorders. In experiments in anesthetized rats, Avlar et al. provide a characterization of how ctACS alters cerebellar neuronal activity and the pattern of response of vestibular neurons.

In summary, this second volume highlights the cerebellum's broad influence on behavior in health and disease through its interconnectivity with the rest of the central nervous system. This diversity of function may be rooted in the construction and updating of internal models through processing of prediction error across the motor and non-motor domains.

## Author contributions

RA: Writing – original draft, Writing – review & editing. JF: Writing – original draft, Writing – review & editing. LM: Writing – original draft, Writing – review & editing. TW: Writing – original draft, Writing – review & editing.

